# Association of serum 25-hydroxyvitamin D concentrations with sleep phenotypes in a German community sample

**DOI:** 10.1371/journal.pone.0219318

**Published:** 2019-07-05

**Authors:** Ezgi Dogan-Sander, Anja Willenberg, İnci Batmaz, Cornelia Enzenbach, Kerstin Wirkner, Elisabeth Kohls, Roland Mergl, Joachim Thiery, Jürgen Kratzsch, Ulrich Hegerl, Christian Sander

**Affiliations:** 1 Department of Psychiatry and Psychotherapy, University of Leipzig Medical Center, Leipzig, Germany; 2 Institute of Laboratory Medicine, Clinical Chemistry and Molecular Diagnostics, University of Leipzig Medical Center, Leipzig, Germany; 3 LIFE—Leipzig Research Center for Civilization Diseases, University of Leipzig, Leipzig, Germany; 4 Department of Statistics, Arts and Sciences Faculty, Middle East Technical Faculty, Ankara, Turkey; 5 Institute for Medical Informatics, Statistics, and Epidemiology (IMISE), University of Leipzig, Leipzig, Germany; 6 Institute of Clinical Psychology and Psychotherapy, Bundeswehr University Munich, Neubiberg, Germany; Associazione OASI Maria SS, ITALY

## Abstract

**Background:**

Sleep disorders and vitamin D deficiency are among the most common health problems. Few studies investigated the effect of vitamin D on objectively recorded sleep with sound methodological quality and reasonable temporal proximity.

**Objective:**

To investigate the relationship between serum 25-hydroxyvitamin D (25(OH)D) concentrations and objective sleep parameters assessed within close temporal proximity in a population-based sample. It is expected that higher serum 25(OH)D concentrations are associated with 1) better objective sleep outcomes (longer sleep duration, higher sleep efficiency, earlier mid-sleep time) and 2) more positive subjective sleep evaluations.

**Methods:**

A subset of participants (n = 1045) from the LIFE-Adult-Study was analysed. Measurement of serum 25(OH)D vitamin was performed using an electrochemiluminescence immunoassay. Actigraphic assessments were performed using SenseWear Pro 3 devices. The following objective sleep parameters were calculated: total sleep duration, night sleep duration, night sleep efficiency, midsleep time and wake after sleep onset (WASO). Subjective sleep evaluations were assessed via questionnaire (sleep quality (PSQI), daytime sleepiness (ESS)). Data were analysed applying a multiple linear regression model with a stepwise approach.

**Results:**

The regression models revealed significant associations of serum 25(OH)D concentration with night sleep duration and midsleep time. No association was found for total sleep duration and night sleep efficiency. Higher serum 25(OH)D concentration was further associated with shorter WASO in males but longer WASO in females. Moreover, serum 25(OH)D concentration did not show any significant association with subjective sleep quality and daytime sleepiness.

**Conclusion:**

The results indicate that a higher concentration of serum 25(OH)D is associated with longer and earlier night sleep. Although the present study was able to demonstrate an association between serum 25(OH)D concentration and objective sleep parameters, no conclusion about underlying mechanisms or causal inferences can be drawn.

## Introduction

Vitamin D is being considered more than just a steroid hormone which plays a central role in calcium-phosphorus and bone homeostasis [1,2]. Recently, other effects of vitamin D on different medical conditions such as cardiovascular diseases, autoimmune diseases, depression, diabetes mellitus and obesity have been demonstrated in several studies [3,4,1,5,6]. Locating several extra-skeletal vitamin D receptors in most tissues and cells (e.g., brain, skin) also supports the hypotheses regarding these effects [7,8]. Additionally, vitamin D is found to take a part in brain development and function [9]. Against the background of the wide spread vitamin D deficiency across Europe [10] investigations on the associations between serum vitamin D concentration as well as vitamin D intake and medical conditions are rapidly emerging.

It is hypothesized that vitamin D has a potential role in the regulation of sleep [11,12]. Sleep disorders are also widely common [13]: The prevalence of sleep disorders ranges from 25% to 32.1% depending on study population and assessment methods [14–16]. Sleep disorders were also considered to play a role in the aetiology of many other disorders such as hypertension [17], heart diseases [18] and diabetes [19]. Furthermore, it has been shown that adults with shorter sleep duration exhibit more health risk factors, e.g. obesity, physical inactivity, smoking and alcohol consumption [20]. Since 2009 there has been an increase regarding studies focusing on the relationship between serum vitamin D concentration (as well as vitamin D intake) and different sleep parameters [21]. For instance, a positive association between serum 25-hydroxyvitamin D (25(OH)D) concentrations and self-reported daily sleep duration was demonstrated among elderly adults [22]. Contrary, Shiue (2013) found no relationship between sleep duration and serum 25(OH)D concentrations; but reported a significant inverse association between sleep onset latency (minutes to fall asleep) and serum 25(OH)D concentrations [23]. Additionally, an inverse correlation between Epworth Sleepiness Scale (ESS) scores, indicating daytime sleepiness, and serum 25(OH)D concentration was reported for patients without vitamin D deficiency (25(OH)D >20 ng/mL) but no correlation between ESS scores and serum 25(OH)D concentrations was found in patients with actual vitamin D deficiency [24]. Surprisingly, a direct correlation between ESS scores and serum 25(OH)D levels was reported in black patients with vitamin D deficiency. Beydoun et al. (2014) [25] also reported a significant inverse relationship between serum 25(OH)D concentrations and a factor analytically derived sub-scale of a sleep questionnaire reflecting sleepiness. However, only a marginal negative association was reported between serum 25(OH)D concentrations and very short sleep, when a sub-group with very short sleep duration (<5 hours per night) was compared to a sub-group with normal sleep duration (7–8 hours per night). Among Korean workers an odds ratio of 1.43 for having poor sleep quality (PSQI (Pittsburgh Sleep Quality Index) score<5) was found in case of vitamin D deficiency (<10 ng/mL) [26].

In the current literature there are only few studies using objective sleep parameters (measured by actigraphy or polysomnography) to investigate the relationship between serum 25(OH)D concentrations as well as vitamin D intake and sleep parameters. In a study by Grandner et al. [27] the association between vitamin D intake and subjective as well as objective sleep parameters in postmenopausal women was investigated, resulting in positive correlations between vitamin D intake and sleep acrophase (“the peak of a fitted 24-hour cosine”) as well as self-reported number of naps. No significant correlations were reported between vitamin D intake and total sleep time, sleep efficiency and duration of naps.

In a study among men aged 68 years or older, it was reported that lower serum 25(OH)D concentrations were associated with short sleep time (<5 hours) and low sleep efficiency (<70%) [28]. Despite the association between lower serum 25(OH)D concentrations and wake after sleep onset (WASO) in age-adjusted models, no association was found in multivariable adjusted models. Another study reported shorter sleep duration, higher ESS scores, higher apnoea-hypopnoea-index (AHI) and lower proportion of time in REM to be associated with serum 25(OH)D concentration<20 ng/ml in an unadjusted model, yet in adjusted models only the association between serum 25(OH)D deficiency with shorter sleep time and lower proportion of REM remained significant [29]. In 2017, Piovezan et al. [30] reported a significant association between the risk of 25(OH)D deficiency (serum 25(OH)D concentrations < 30 ng/mL) and short sleep duration (<6 hours) measured by polysomnography. The authors also reported an association between moderate to severe obstructive sleep apnoea and risk of serum 25(OH)D deficiency. In a recent meta-analysis discussing the association between vitamin D deficiency and sleep disorders an inverse relationship between vitamin D levels and risks of sleep disorders (poor sleep quality, short sleep duration and sleepiness) was summarized [31]. Not only sleep parameters but also specific sleep disorders such as narcolepsy have been associated with low serum levels of vitamin D [32].

Taken together, there are some hints on lower serum 25(OH)D concentrations being associated with poorer subjective and objective sleep outcomes. However, several methodical limitations within the existing studies need to be considered. For example, in the study by Bertisch et al. [29] there was an average time gap of 10.3 years between serum 25(OH)D measurements and collection of sleep outcomes whereas the study population of Massa et al. [28] comprised only of older men. Considering the importance of this research field and the subsequent public health relevance, we aimed to enlighten the complex link between two of the most common health problems—sleep disorder and vitamin D deficiency–by investigating the relationship between serum 25(OH)D concentrations and objective and subjective sleep parameters assessed within close temporal proximity in a population comprising of both genders and a broader age span. Based on the available literature we expected higher serum 25(OH)D concentrations to be associated with

1. better objective sleep (longer sleep duration, higher sleep efficiency, earlier mid-sleep time); and
2. more positive subjective sleep evaluations (better subjective sleep quality and less subjective daytime sleepiness).

## Materials and methods

### Database and study population

The presented data stem from the large-scale research project ‘LIFE’ (Leipzig Research Center for Civilisation Diseases). Within the LIFE-Adult-Study, a population-based sample of 10.000 participants (18 to 79 years) was recruited between August 2011 and November 2014 in Leipzig, Germany [33]. All subjects gave written informed consent to participate in the examinations. The procedures were conducted according to the Declaration of Helsinki and approved by the University of Leipzig Medical Faculty's ethics committee (registration-number: 263-2009-14122009). One of the aims of the LIFE project was to assess the prevalence and incidence of common diseases (cardiovascular diseases, metabolic diseases, cognition and brain functioning, depression, sleep disorders, degenerative diseases of the retina, allergies and immune competence) and subclinical disease phenotypes. In addition to standardized interviews (e.g. for sociodemographic information and medical history), several physical and medical examinations (e.g. anthropometric measurements) as well as laboratory tests (e.g. blood sampling) were performed. A random subgroup of LIFE-participants wore an actigraphy device for seven consecutive days.

**Fig 1:**
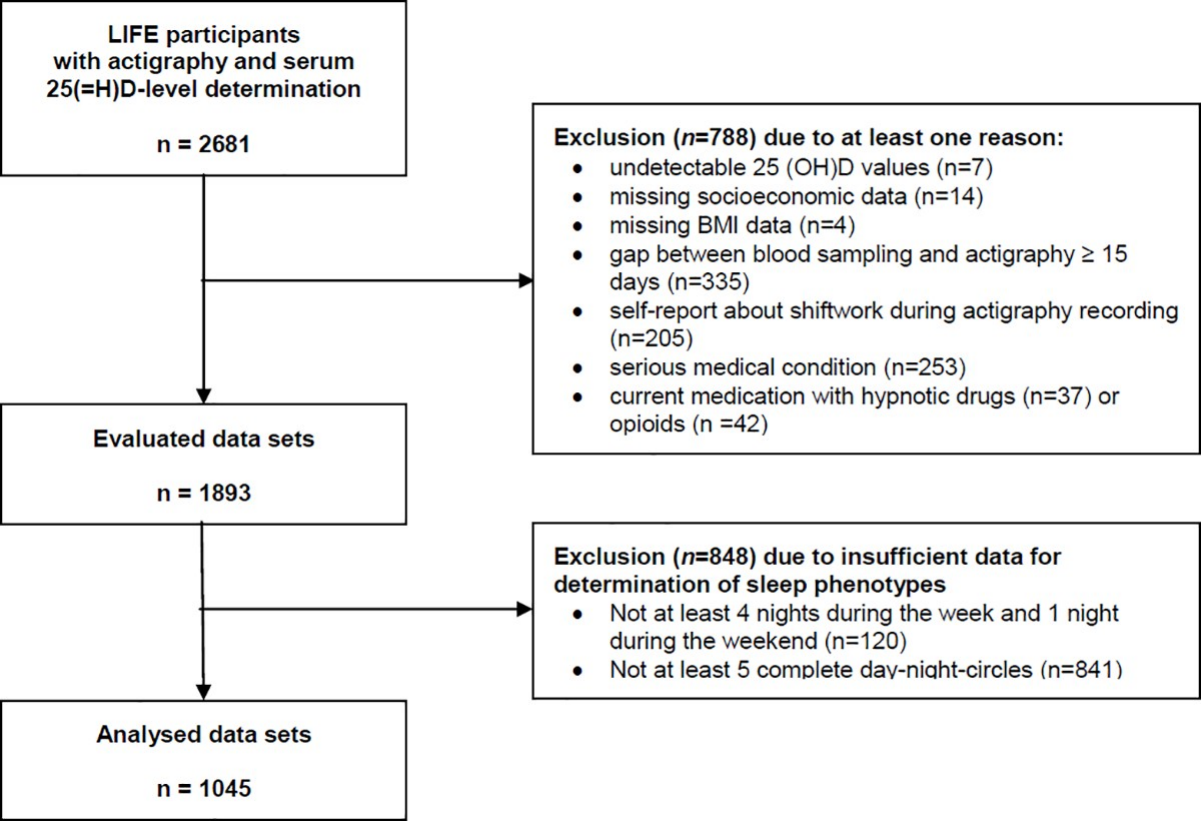
Flow diagram illustrating the data selection process.

For the present investigation, we analysed participants from the LIFE-sample for whom actigraphy recording existed and serum 25(OH)D concentrations had been measured. From this total sample of 2681 participants, 788 were excluded according to at least one of the following criteria (see also Fig 1):

- undetectable serum 25(OH)D values (N = 7);
- missing socioeconomic data (N = 14);
- missing BMI measurement (N = 4);
- more than 15 days between blood collection for 25(OH) D measurement and actigraphy monitoring (N = 335);
- reported shift-work during the actigraphy period (N = 205);
- serious medical conditions (N = 253 participants with either a lifetime history of: Parkinson syndrome, intermittent claudication, autoimmune diseases, Crohn`s disease, ulcerative colitis, renal insufficiency, multiple sclerosis, requiring dialysis, liver cirrhosis, HIV; or within the last 12 month incidence of: cancer, myocardial infarction, stroke, tuberculosis, hepatitis and highly dangerous alcohol consumption (>80 gr/day for women, >120 gr/day for men)),
- medication with sedative-hypnotic drugs (N = 37) or opioids (N = 42) at the day of blood sampling.

Of the remaining 1893 participants, 848 participants had to be excluded due to their actigraphy data not meeting the requirements for evaluation (see methods section below). The final sample (N = 1045) comprised of 512 males and 533 females.

### Assessment procedures

#### Serum 25(OH)D analyses

Blood withdrawal was performed according the protocol [33]. Measurement of serum vitamin D total was performed according to manufacturer’s protocol on an automated laboratory analyzer Cobas 8000 e602 (Roche Diagnostics, Mannheim Germany) using an electrochemiluminescence immunoassay (ECLIA) with competition principle (Roche Diagnostics Mannheim Germany). Traceability of the method was standardized against LC-MS/MS [34]and LC-MS/MS in turn was standardized against the NIST standard [35]. According to manufacturer information the limit of quantitation is 5 ng/ml. The primary measurement range is 5–70 ng/ml. Samples with concentration higher than 70 ng/ml were diluted manually 1:2 with Diluent Universal (Roche Diagnostics, Mannheim, Germany)

Between October 2011 and November 2014 VK of control level 1 varied between 6% and 17.5% (mean 10.13%), control level 2 varied between 3.4% and 12.2% (mean 6.89%). For the main regression analyses, we used the detected serum 25(OH)D concentrations as continuous variable. However, for additional analyses (see S2 Table) we subdivided the sample into groups based on recommendations from the literature [36,37,31,12]: vitamin D deficiency (≤10 ng/ml), mild vitamin D deficiency (>10–20 ng/ml), vitamin D insufficiency (>20–30 ng/ml) and sufficient amount (>30 ng/ml).

#### Actigraphy

Actigraphic assessments in LIFE were performed using SenseWear Pro 3 devices which recorded multiple sensor parameters (2-axis body acceleration, skin temperature, heat flux, galvanic skin response) and automatically detects removal of devices (off-arm detection). Participants were asked to wear the devices for one week and to keep a sleep diary concerning activity and bedtime periods (‘lights off time’ and ‘get up time’). Basic analyses were performed using the SenseWear Professional 6.1 software package (BodyMedia; Pittsburgh, Pennsylvania), which uses specific algorithms to classify 1min-periods as either being up versus lying down (hereafter called , rest‘) and wake versus asleep (hereafter called ‚sleep‘). The SenseWear was successfully validated against polysomnography, showing accurate detection of total sleep time, sleep efficiency and wake after sleep onset [38–42] as well as sleep onset and sleep offset [43,44].

After automatic sleep and rest classification, scored data was exported to a Microsoft Excel template with Visual Basic for Applications (VBA) macros, which was used to compute variables not calculated by the SenseWear software (e.g. wake after sleep onset). Additionally, the Excel tool was used to customize analysis windows to the specific day-night-cycles of each participant, as no fixed time-window was used for night or day-sleep analyses. Based on the bedtime information provided in the sleep diary the respective sleep/rest episodes belonging to a night sleep interval (NSI) were identified manually. Afterwards, “days” were defined as spanning from the midpoint of a NSI to the midpoint of the following NSI and „daytime”was defined as the period between two NSIs. A daytime interval (DTI, defined as the period between two consecutive NSIs) therefore contains all sleep/rest episodes that were tagged outside of a NSI. For analyses concerning night sleep, all NSIs containing off-arm periods were excluded. For day sleep analyses, only ‚days‘ with a minimum duration of 20 hours and an off-wrist duration of less than 15% of the DTI were used. Total sleep duration was calculated by summing up nighttime sleep duration and sleep duration during the following day (night-day-cycles = NSI + DTI). The following sleep parameters were than calculated based on the rest and sleep classification by the SenseWear software:

- Total sleep duration (TSD; sum of all minutes classified as sleep during a night-day- cycle (NSI + DTI));
- Night Sleep duration (NSD = sum of all minutes classified as sleep during the NSI);
- Night sleep efficiency (NSE = ratio of NSD to total sum of all minutes classified as lying down during the NSI);
- Midsleep time (MST = half-time between sleep onset and sleep offset as detected by the SenseWear algorithm within the NSI);
- Wake after sleep onset (WASO; sum of all minutes classified as wake between sleep onset and offset).

For further analyses, daily actigraphic results were individually averaged across the respective measuring days. Data sets were excluded from further analyses when they did not match the following standards: a) at least four week nights and at least one weekend night, and b) at least 5 night-day-cycles (see also Fig 1). Therefore, averaged sleep variables are based on at least five nights/night day-circles including at least 1 weekend night/night-day circle.

#### Questionnaires & interviews

During the baseline visit in the LIFE study centre information on sociodemographic and socioeconomic factors, medical history, current medication and subjective well-being were collected using questionnaires and standardized interviews performed by trained raters. Based on information on education (school, professional), occupational status, and equivalent household income a multidimensional index of socioeconomic status was calculated [45,46]. Body weight measured using an electronic scale and body height assessed by means of a stadiometer were used to calculate body mass index. Information on medical history was obtained by asking for occurrence of specific diseases (lifetime occurrence, presence in the last 12 months and current treatment). Medication taken in the last seven days before the baseline visit (including vitamin D supplements) was registered via ATC (Anatomical Therapeutic Chemical) classification system for data analysis. Alcohol consumption within the last year was assessed by inquiring the amount and frequency of alcoholic beverages [33]. Participants were asked to evaluate their daytime sleepiness using the Epworth Sleepiness Scale (ESS) [47]. Subjective sleep quality was assessed using the Pittsburgh Sleep Quality Index (PSQI) [48].

### Statistical analyses

Given the limited missing data (for each covariate < 1.7% missings) a complete-case analysis had been performed. Multiple linear regression (MLR) models of the following form were fitted:

$$y=\beta_{0}+\beta_{1}x_{1}+\cdots +\beta_{k}x_{k}+\varepsilon$$ (1)

Y represents the response and Χ represents the independent variables of interest; k is the total number of independent variables involved in (1); ε is the error term. β_i_ are unknown regression coefficients to be estimated.

The response variables are the objective (1. total sleep duration, 2. night sleep duration, 3. night sleep efficiency, 4. midsleep time, 5. wake after sleep onset) and subjective sleep outcomes (6. sleep quality (PSQI score) and 7. daytime sleepiness (ESS Score)). The **independent variables** are serum 25-(OH)D concentration and the following covariates: age, gender, marital status, BMI, socioeconomic status, alcohol consumption, histories of lifetime thyroid disorder and lifetime depression, vitamin D intake, employment status and seasons. Categorical variables had been converted to dummy variables to be used in the models (as a result, dummy employed and unemployed were compared to being retired for the employment status; low and middle were compared to high socioeconomic status; being married, being single, being divorced, being married but living apart were compared to being widow for marital status and summer, winter and spring were compared to fall).

In order to obtain reliable results from the analysis, the plausibility of the basic assumptions for the MLR models was assessed [49,50]. These assumptions include: a) the model given in Eq (1) adequately explains the relationship between the response and independent variables and b) the error term, ε, is distributed normally with zero mean and constant variance. For checking normality Shapiro-Wilk test (the most powerful test for normality) or Kolmogorov-Smirnov (K-S) test was used. For the same purpose, normal probability plots (NPP) of standardized residuals were also examined. Outliers were detected and treated where necessary (see below). Because there were no replicated observations in data, homogeneity assumption of errors was evaluated by the standardized residuals versus the fitted values plot. This plot is also useful in detecting inadequacy of the model fitted, for which an approximate statistical test was also conducted. Furthermore, there are some measures which diagnose if there is an unduly effect of an observation on the fitted model, called ‘influential observation‘. To label an observation as influential, we adopted a rather conservative approach: In each analysis we examined the diagnostic measures cook`s distance (D), leverage (h), difference in fits (DFFITS) and covratio (COV). An observation was excluded from the analysis if at least three of these diagnostic measures were identified as significant.

In this study, the following strategy had been adopted while developing the final regression models [49,50]: The initial MLR models included serum 25-hydroxy vitamin D levels and all covariates. When the non-normal error distribution was symmetric but its non-normality was due to the longer tails than normal distribution, outlier observations (whose standardized residuals were out of [–3, 3] range) were removed from the analysis or otherwise Box-Cox transformation was applied on the response, and the model was refitted. In case of influential observations, these were excluded and the model was again refitted.

Because of the high number of variables involved in the initial models, multicollinearity is unavoidable. Therefore, a stepwise regression method was used to determine the most significant variables. MLR models were refitted only with the significant variables. If lack-of-fit was detected, higher order model terms involving interactions of variables were included in the model. Sometimes, these terms also caused another multicollinearity problem in the analysis. To overcome this problem, a centring approach was utilized, where the variable X is replaced with (X-average(X)), and the centred X variable was labelled as X_C (i.e. X variable centred). The **final models** were obtained, once all assumptions of least square regression were satisfied.

All analyses were conducted by using the statistical software SPSS 24 (IBM SPSS Statistics for Windows, version 24 (IBM Corp., Armonk, N.Y., USA)) and Minitab 14 (Minitab version 14, Minitab Inc., State College, PA, USA). Also, the significance level was taken to be α = 0.05 (two-tailed).

## Results

The sociodemographic characteristics of the final sample are shown in Table 1, while Table 2 depicts the descriptive results concerning sleep parameters and serum 25(OH)D assessment. Differences in sleep phenotypes between the four vitamin D groups (Vitamin D deficit, shortage, insufficiency and sufficiency) are presented in S1 Table.

**Table 1:** Sociodemographic and medical characteristics of the study population.

Characteristics	Total Sample	Males	Females
N	1045	512 (49.0%)	533 (51.0%)
Age (years), mean (SD)	59.07 (11.68)	59.79 (11.71)	58.38 (11.62)
Age-Groups			
18–39 years	36 (3.4)	20 (3.9)	16 (3.0)
40–49 years	214 (20.5)	90 (17.6)	124 (23.3)
50–59 years	254 (24.3)	119 (23.2)	135 (25.3)
60–69 years	310 (29.7)	159 (31.1)	151 (28.3)
70+ years	231 (22.1)	124 (24.2)	107 (20.1)
Marital Status, n (%)			
Married, not living separately	684 (65.5)	365 (71.3)	319 (59.8)
Married, but living separately	26 (2.5)	7 (1.4)	19 (3.6)
Single	130 (12.4)	71 (13.9)	59 (11.1)
Divorced	137 (13.1)	53 (10.4)	84 (15.8)
Widowed	68 (6.5)	16 (3.1)	52 (9.8)
Socioeconomic status, n (%)			
Low	163 (15.6)	78 (15.2)	85 (15.9)
Middle	634 (60.7)	295 (57.6)	339 (63.6)
High	248 (23.7)	139 (27.1)	109 (20.5)
Employment status, n (%)			
Employed	518 (49.6)	245 (47.9)	273 (51.2)
Unemployed	73 (7.0)	38 (7.4)	35 (6.6)
Retired	454 (43.4)	229 (44.7)	225 (42.2)
BMI (kg/m^2^), mean (SD)	27.73 (4.52)	27.96 (3.86)	27.51 (5.06)
BMI groups, n (%)			
underweight (<18.5)	4 (0.4)	1 (0.2)	3 (0.6)
normal weight (18.5–24.99)	285 (27.3)	108 (21.1)	177 (33.2)
overweight (25.0–29.99)	484 (46.3)	271 (52.9)	213 (40.0)
obese (30 and higher)	272 (26.0)	132 (25.8)	140 (26.3)
Alcohol consumption (g/day)^a^, mean (SD)	11.77 (16.23)	17.66 (19.80)	6.03 (8,50)
Vitamin D Intake, n (%)	193 (18.5)	71 (13.9)	122 (22.9)
Lifetime depression diagnosis, n (%)	101 (9.7)	30 (5.9)	71 (13.3)
Lifetime thyroid disorder diagnosis, n (%)	267 (25.6)	67 (13.1)	200 (37.5)

Annotations: SD = standard deviation, BMI = Body Mass Index

^a^ reduced n due to missing values: alcohol consumption (total: N = 1027, males: N = 507, females: N = 520)

**Table 2:** Sleep parameters and serum 25(OH)D concentrations of the total sample separated by gender.

Characteristics	Total Sample	Males	Females
N	1045	512 (49.0%)	533 (51.0%)
Serum 25(OH)D-Concentration (ng/ml), mean (SD)	21.94 (10.65)	22.08 (10.71)	21.80 (10.60)
Total sleep duration (min), mean (SD)	401.27 (63.55)	389.27 (66.12)	412,80 (58,77)
Night sleep duration (min), mean (SD)	379.27 (58.17)	366.42 (59.70)	391.61 (53.92)
Night sleep efficiency (%), mean (SD)	82.36 (8.17)	80.84 (8.93)	83.83 (7.08)
WASO (min), mean (SD)	70.24 (42.04)	75.43 (46.15)	65.24 (37.03)
Midsleep time (hh:mm), mean (SD)	3:08 (0:49)	3:12 (0:52)	3:04 (0:46)
PSQI Score ^a^, mean (SD)	5.20 (3.19)	4.49 (2.74)	5.89 (3.43)
ESS Score ^a^, mean (SD)	7.89 (3.50)	8.29 (3.51)	7.51 (3.45)

Annotations: SD = standard deviation, WASO = Wake after Sleep Onset, PSQI = Pittsburgh Sleep Quality Index, ESS = Epworth Sleepiness Scale

^a^ reduced n due to missing values: PSQI-score (total: N = 985, males: N = 485, females: N = 500), ESS Score (total: N = 1014, males: N = 500, females: N = 514)

Linear regression analyses were performed for all sleep outcomes to investigate the associations between serum 25(OH) levels and sleep phenotypes. For each sleep phenotype, non-significant variables were excluded for further analyses with a stepwise approach. Results of the final regression models are described in the following sections (for a summary of the ANOVA results of the final model built see S2 Table).

### Vitamin D and objective sleep parameters

#### Total Sleep Duration (TSD)

According to the regression analysis results, no statistically significant relation was detected between TSD and serum 25(OH)D concentrations (β = 0.042; p = .208). However, the following variables were found to be significantly associated with TSD (see also Table 3): Higher BMI (p = .008), male gender (p< .001), being employed (p = .002) and season summer (p = .015) were associated with decreased TSD; while higher age (p = .001) and history of lifetime thyroid disorder (p = .021) were associated with increased TSD.

**Table 3:** Results of final multiple linear regression models for objective and subjective sleep phenotypes.

Response variables	N	Independent variables (in the final model)	β	p
Total Sleep Duration	1029	Gender male	-0.184	< .001
		Age	0.139	.001
		Being employed	-0.129	.002
		BMI	-0.081	.008
		Season Summer	-0.072	.015
		LTH thyroid disorder	0.071	.021
Night Sleep Duration (tr)	1019	Serum 25(OH) D concentration	0.065	.038
		Gender male	-0.214	< .001
		Being employed	-0.147	< .001
		Season Summer	-0.072	.019
		BMI	-0.102	.001
		LTH tyhroid disorder	0.058	.066
Night Sleep Efficiency (tr)	1005	Gender male	-0.167	< .001
		Being married	0.112	< .001
		BMI (centered)	-0.150	< .001
		BMI (centered) x Gender male (centered)	-0.091	.004
Midsleep time (tr)	1021	Serum 25(OH) D concentration (centered)	-0.080	.011
		Age (centered)	-0.154	.001
		Low SES (centered)	-0.060	.050
		Alcohol consumption (centered)	0.192	< .001
		Season Spring (centered)	-0.064	.034
		Being employed (centered)	-0.349	< .001
		Age x Gender male	-0.086	.004
		Age x Being single	-0.114	.001
		Gender Male x Alcohol consumption	-0.127	.001
Wake after Sleep Onset (tr)	1044	Serum 25(OH) D concentration	0.082	.048
		LTH thyroid disorder	-0.086	.024
		BMI	0.078	.015
		Gender male x Being employed	-0.136	< .001
		Gender male x BMI	0.258	< .001
		Gender male x LTH thyroid disorder	0.083	.029
		Gender male x Serum 25(OH)D concentration	-0.152	.030
Daytime Sleepiness (ESS-Score)	1010	Gender male	0.130	< .001
		Being employed	0.206	< .001
		Being unemployed	0.066	.039
		LTH depression	0.074	.017
Subjective Sleep Quality (PSQI-Score) (tr)	985	Age	0.139	< .001
		BMI	0.079	.011
		LTH depression	0.132	< .001
		Gender male x Age	-0.288	< .001
		Gender male x LTH thyroid disorder	0.083	.009
		Gender male x Season Winter	0.085	.012

Annotation: β: beta coefficient; tr: Box-Cox transformed data; BMI: Body mass index; SES: socioeconomic status, LTH: Lifetime history

#### Night Sleep Duration (NSD)

**Fig 2:**
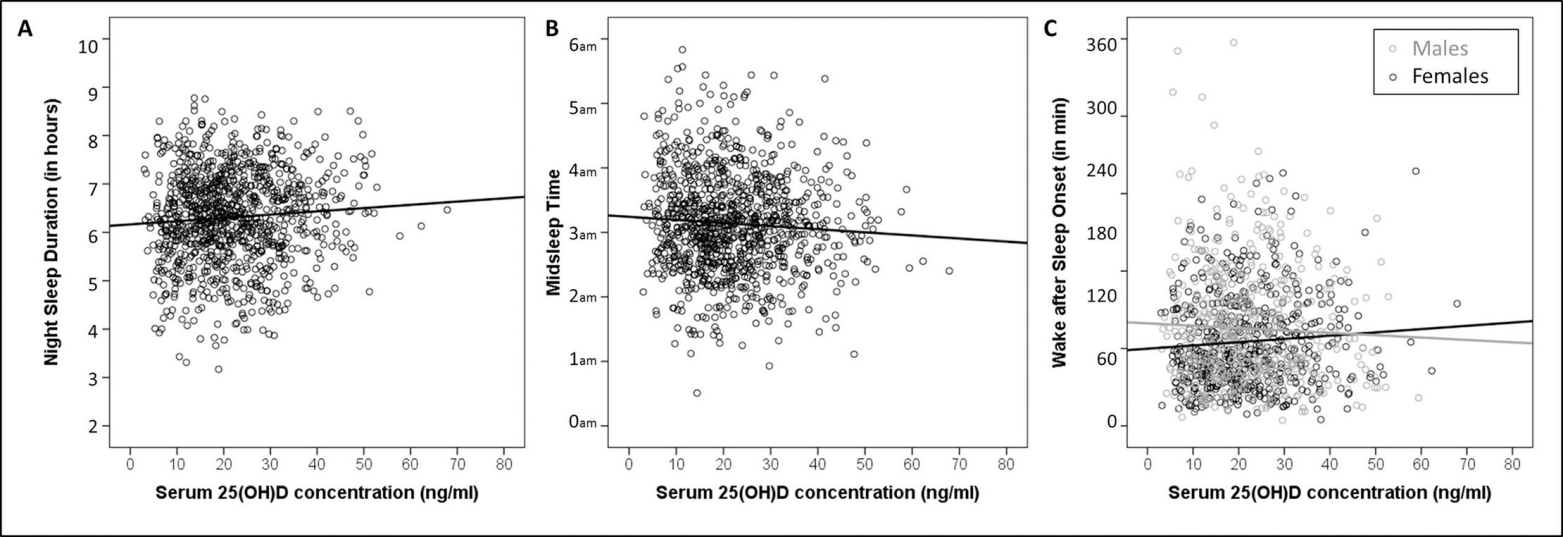
Scatterplots depicting associations between serum 25(OH)D concentration and Night Sleep Duration (A), Midsleep Time (B) and Wake after Sleep Onset (C).

Initially, non-normality of errors was detected. To resolve this problem, we employed a Box-Cox transformation on the night sleep duration values, which resulted in an optimal λ = 1.5. In the final model (see Table 3), higher serum 25(OH)D concentrations were related significantly with longer NSD (β = 0.065; p = .038, see Fig 2A). In addition, male gender (p< .001), being employed (p< .001), season summer (p = .019) and higher BMI (p = .001) showed inverse relationships with NSD whereas lifetime history of thyroid disorder showed a tendency to relate with longer NSD (p = .066).

#### Night Sleep Efficiency (NSE)

The initial analysis indicated a severe left skewness of NSE. As a remedial measure, the response was transformed with optimal λ = 3.70 as suggested by Box-Cox. To overcome the lack-of-fit that existed in the analysis (p-value = 0.035), interaction terms related to centred BMI and centred male gender were also included in the final model (see Table 3). However, no statistically significant relation was detected between NSE and serum 25(OH)D concentrations (β = -0.027; p = .423). Results indicated that only the covariates gender, marital status and BMI have significant relationships with NSE: Being married was related to higher NSE (p< .001) and an inverse relationship between BMI and NSE was found, which was stronger in males than the females (p = .004) (see S1A Fig).

#### Midsleep time (MST)

In the initial MLR model, violation of normality assumption was detected, therefore a Box-Cox transformation was applied with optimal lambda (λ) = 0.74. Additionally, to avoid the multicollinearity the variables were centered. To overcome the lack-of-fit that existed in the analysis interaction terms were also included in the final model (see Table 3). Results indicated that serum 25(OH)D concentration showed an inverse relationship to MST (β = -0.080; p = .011) (see Fig 2B). Low socioeconomic status (p = .050), being employed (p< .001) and season spring (p = .034) showed an inverse relationship to MST. MST also showed a negative association with age in singles (p< .001). Additionally, alcohol consumption was positively (p< .001) and age was negatively related to MST in males (p = .004). For interaction plots see S1B–S1D Fig .

#### Wake after Sleep Onset (WASO)

WASO had a right skewed distribution, which resulted in violation of the normality assumption. As a result, Box-Cox transformation was applied, and the optimal lambda (λ) was found to be 0.23. In addition, there was lack-of-fit of the MLR model studied as well (p-value = 0.024). As a remedial measure, interactions of the covariates being employed, BMI and having lifetime thyroid disorder with gender were also contained in the final model (see Table 3). According to the results, higher serum 25(OH)D concentration was associated with shorter WASO in males and longer WASO in females (p = .030) (see Fig 2C). In males, higher BMI (p< .001) and positive lifetime thyroid disorder (p = .029) were related to more WASO. Additionally, being employed showed negative relationship with WASO in males (p< .001). Interaction plots are shown in S1E–S1G Fig.

### Vitamin D and subjective sleep evaluations

#### Daytime sleepiness (ESS score)

In the final model (see Table 3), no statistically significant relation was detected between ESS score and the serum 25(OH)D concentrations (β = 0.031; p = .396). However, male gender (p< .001), being employed (p< .001) as well as being unemployed (p = .039) and lifetime depression history (p = .017) showed positive relationships with daytime sleepiness.

#### Subjective sleep quality (PSQI score)

Analysis of residuals showed that PSQI scores had a right skewed distribution. The optimal lambda, λ = 0.26, was determined by Box-Cox transformation. In the final model (see Table 3) the following interaction terms were included: gender *age, gender * lifetime thyroid disorder and gender * season winter. Still, no statistically significant relationship was detected between PSQI scores and serum 25(OH)D concentrations (β = -0.015; p = .666). Higher PSQI scores, indicating worse subjective sleep quality, were related to higher BMI (p = .011) and positive history for lifetime depression (p< .001). Additionally, in males higher PSQI-scores were associated with lower age (p< .001), positive lifetime thyroid disorder history (p = .009) and season winter (p = .012) (see S1H–S1J Fig for interaction plots).

## Discussion

This study aimed to investigate the relationship between serum 25(OH)D concentrations and objective and subjective sleep parameters assessed within close temporal proximity. As hypothesized, this study demonstrated a link between serum 25(OH)D concentrations and objectively recorded sleep parameters. On the other hand, no association between subjective sleep evaluations and 25(OH)D concentrations was found. Although serum 25(OH)D concentrations did not show associations with total sleep duration or night sleep efficiency, a positive correlation between night sleep duration and serum 25(OH)D concentrations as well as an inverse association between midsleep time and serum 25(OH)D concentrations were found. This indicates that a higher concentration of serum 25(OH)D concentration is associated with longer and earlier night sleep. Furthermore, higher levels of serum 25(OH)D were associated with shorter wake phases after sleep onset (WASO) in males but longer WASO in females.

These results are mainly in line with results of previous studies, where sleep had been assessed objectively using actigraphy and a positive relationship between serum 25(OH)D concentrations and night sleep duration had been reported [28–30]. However, those studies focused on night sleep alone and did not assess total sleep duration (i.e. the total amount of sleep during the night and the day). Therefore our non-significant results concerning total sleep duration are not comparable. Bertisch et al. [29] reported a significant relationship between night sleep efficiency and serum 25(OH)D concentrations from an unadjusted model, but not in adjusted models. Contrary to the results from the current study, Massa et al. [28] and Piovezan et al. [30] reported a relationship between lower concentrations of serum 25(OH)D and poor night sleep efficiency. To our knowledge, the association of 25(OH)D concentration and WASO has not been investigated in mixed-gender populations. Massa et al. [28] reported an inverse association between serum 25(OH)D concentrations and WASO in a sample comprising only of male subjects. Their result is consistent to our finding of higher levels of serum 25(OH)D concentration being associated with shorter WASO in male subjects. The association between higher serum 25(OH)D concentrations and longer WASO in females warrants further replication.

Contrary to our hypotheses, no associations were found between serum 25(OH)D concentration and subjective sleep evaluations (sleep quality according to PSQI, daytime sleepiness according to ESS). Inconsistent results had been reported in studies applying self-reported sleep duration, assessed via questionnaires and sleep logs. Kim et al. [22] reported a positive relationship between serum 25(OH)D concentrations and sleep duration, whereas Shiu [23] found no significant association. In contrast to our results, Jung et al. demonstrated a correlation between 25(OH)D levels and sleep quality using PSQI [26]. While Beydoun et al. [25] reported an inverse relationship between serum 25(OH)D concentration and sleepiness (i.e. one of three factors extracted by an exploratory factor analytic approach from sleep-related scale items of a comprehensive interview of the NHANES Survey). Based on the same survey data, Shiu [23] highlighted that lower serum 25(OH)D concentration were on the other hand associated with longer self-reported minutes to fall asleep while people with higher serum 25(OH)D concentration reported more sleep complaints. Methodological differences might explain these inconsistent findings. Indeed, results of the current study do corroborate the finding of Bertisch et al. [29] who also used the ESS to assess sleepiness. Still, no significant association between ESS scores ad serum 25(OH)D concentration could be found in an adjusted regression model. 25(OH)D deficiency was only related to higher ESS scores in unadjusted models.

The present study was able to demonstrate an association between serum 25(OH)D concentration and objective sleep parameters. However, no conclusion about the mechanisms underlying such an association can be derived. There is growing evidence regarding the physiological processes involved in the regulation of sleep that allow some cautious assumptions. Sleep is regulated by homeostatic regulation as well as a circadian clock [51]. The central circadian clock is located in suprachiasmatic nuclei of the anterior hypothalamus. Additionally, the subparaventricular zone and the dorsomedial nucleus of the hypothalamus were found to be involved in the regulation of sleep/wakefulness. By causing arousal, the posterior hypothalamus has a wakefulness-promoting influence. It has also been shown that enzymes controlling vitamin D activation and degradation are expressed in the brain which indicates that the brain can synthesize its own vitamin D [52]. The presence of vitamin D receptors in different brain regions such as anterior and posterior hypothalamus and substantia nigra [7] supports the hypothesis of the complex link of vitamin D and sleep patterns [11]. Furthermore, there is evidence that vitamin D can affect brain serotonin concentration and in this way regulate the production of melatonin which controls circadian rhythms and sleep [52,53]. Nonetheless, no causal conclusions can be drawn from the association studies, as mentioned before. Sunlight exposure and nutritional differences could impact both serum 25(OH)D levels and sleep and it has been discussed whether longer sleep duration may also reduce the opportunity of sunlight exposure and to eat vitamin D rich foods which may also cause vitamin D deficiency. However, in our study shorter sleep duration was associated with lower serum 25(OH)D levels. At present only few studies have investigated the impact of vitamin D supplementation on sleep behaviour with inconsistent results. An improvement of all sleep outcomes (global PSQI score, sleep latency, sleep duration and sleep efficiency) after vitamin D supplementation was shown in veterans with chronic pain [54]. On the other hand an increased need for sleep medication and worse sleep quality after vitamin D supplementation were reported in overweight or obese postmenopausal women [55].

The present study has some limitations, which need to be considered: Although the statistical approach did control for covariates such as BMI, alcohol consumption, lifetime thyroid disorder or season, it is still possible that other potential confounders influenced the sleep outcomes. Also, 18.5% of the sample was found to be taking vitamin D supplements. We did not exclude those subjects but included intake of vitamin D supplements as a covariate. However, the available information on vitamin D intake was restricted to pharmaceutical products, thus not considering food consumption or prescription free nutritional supplements. A potential bias due to that reason is possible.

On the other hand this study had a number of major strengths, especially compared to the existing literature body in this research area. The investigated study population is way larger and not restricted to a certain gender or age, thus improving the external validity and interpretability in comparison with previous studies. Also, the time lag between measurement of serum 25(OH)D levels and assessment of sleep outcomes was kept relatively short (± 15 days), thus improving the general validity of the study results. Second, we controlled for multiple potential confounders and found complex associations with serum 25(OH)D concentrations and several objective sleep parameters. To further enlighten the complex link between vitamin D and sleep more studies in this field are necessary, especially focusing on a combination of subjective and objective assessments of sleep parameters.

## Supporting information

S1 Table Sleep Phenotypes in the total sample and subgroups according to serum 25(OH)D-levels. (DOCX)

S2 Table ANOVA results of the final models built for the respective sleep phenotypes. (DOCX)

S1 Fig Interaction plots for significant associations between selected sleep phenotypes and co-variables within the multiple linear regression models. (TIF)

S1 Data Minimal anonymized data set necessary to replicate study findings. (XLSX)
