# Supplementary material for: Association of serum 25-hydroxyvitamin D concentrations with sleep phenotypes in a German community sample
Source: PLoS One. 2019 Jul 5;14(7):e0219318. doi: 10.1371/journal.pone.0219318 (PMC6611612; doi:10.1371/journal.pone.0219318)
Supplement: S1 Table — (DOCX) [file pone.0219318.s001.docx]

S1 Table: Sleep Phenotypes (Means ± SD) in the total sample and subgroups according to serum 25(OH)D-levels: deficit (≤ 10 ng/ml); shortage (>10-20 ng/ml); insufficiency (>20-30 ng/ml) and sufficient vitamin D (> 30ng/ml)

|  | Total Sample  (n = 1045) | Deficit  (n = 128) | Shortage  (n=382) | Insufficiency  (n=318) | Sufficiency (n=217) | Kruskal Wallis |
| --- | --- | --- | --- | --- | --- | --- |
| Gender (males/females) | 512/533 | 63/65 | 191/191 | 149/169 | 109/108 | Chi² = 0.872  p = .832 |
| Age (in years) | 59.07 ± 11.68 | 56.06 ± 11.89 | 57.72 ± 11.77 | 60.21 ± 11.37 | 61.55 ± 11.20 | Chi² = 25.517  p < .001 * |
| Total sleep duration  (in min) | 401.27 ± 63,55 | 388,37 ± 64,52 | 403,00 ± 62,22 | 399,70 ± 65,25 | 408,13 ± 61,98 | Chi² = 10,741  p = .013 * |
| Night sleep duration  (in min) | 379.27 ± 58.17 | 363.78 ± 56.64 | 381.70 ± 57.62 | 377.51 ± 59.09 | 386.69 ± 57.26 | Chi² = 15.157  p = .002 * |
| Night sleep efficiency  (in %) | 82.36 ± 8.17 | 81.76 ± 8.61 | 82.93 ± 8.03 | 81.63 ± 8.41 | 82.80 ± 7.76 | Chi² = 3.953  p = .267 |
| Wake after sleep onset  (in min) | 70.24 ± 42.04 | 72.07 ± 47.80 | 67.48 ± 41.11 | 74.04 ± 43.03 | 68.43 ± 38.18 | Chi² = 4.254  p = .235 |
| Midsleep time  (HH:MM) | 3:08 ± 0:50 | 3:15 ± 0:54 | 3:07 ± 0:52 | 3:07 ± 0:48 | 3:06 ± 0:43 | Chi² = 3.549  p = .314 |
| Subjective Sleep Quality (PSQI Score) | 5.20 ± 3.19 | 5.24 ± 3.15 | 5.20 ± 3.28 | 5.30 ± 3.20 | 5.02 ± 3.04 | Chi² = 1.417  p = .702 |
| Daytime Sleepiness  (ESS Score) | 7.89 ± 3.50 | 8.11 ± 3.80 | 7.70 ± 3.30 | 8.11 ± 3.63 | 7.78 ± 3.45 | Chi² = 2.212  p = .530 |

Annotations: Reduced sample size due to missing data in questionnaires (PSQI: n = 985 (125/358/301/201); ESS: n=1014 (124/369/309/212)

* Post-hoc-Analyses (Mann-Whitney-Test):

- Age: deficit vs. shortage: p= .147 / deficit vs. insufficiency: p= .001 / deficit vs. sufficiency: p< .001, shortage vs. insufficiency: p= .005 / shortage vs. sufficiency: p< .001, insufficiency vs. sufficiency: p= .203
- Total Sleep Duration: deficit vs. shortage: p= .008 / deficit vs. insufficiency: p= .025 / deficit vs. sufficiency: p= .001, shortage vs. insufficiency: p= .823 / shortage vs. sufficiency: p= .243, insufficiency vs. sufficiency: p= .213
- Night Sleep Duration: deficit vs. shortage: p= .001 / deficit vs. insufficiency: p= .012 / deficit vs. sufficiency: p< .001, shortage vs. insufficiency: p= .523 / shortage vs. sufficiency: p= .266, insufficiency vs. sufficiency: p= .110
