# Supplementary material for: Association of serum 25-hydroxyvitamin D concentrations with sleep phenotypes in a German community sample
Source: PLoS One. 2019 Jul 5;14(7):e0219318. doi: 10.1371/journal.pone.0219318 (PMC6611612; doi:10.1371/journal.pone.0219318)
Supplement: S2 Table — (DOCX) [file pone.0219318.s002.docx]

S2 Table: ANOVA results of the final models built for the respective sleep phenotypes.

| **Variable** | **Source** | **SS** | **df** | **MS** | **F** | $\boldsymbol{p}$**-value** | **R²** | **R²_adj_** |
| --- | --- | --- | --- | --- | --- | --- | --- | --- |
| Total Sleep Duration (TSD) | Regression | 1.490 E9 | 6 | 2.483 E8 | 20.133 | <.001 | 0.106 | 0.100 |
|  | Residual Error | 1.260 E10 | 1022 | 1.233 E7 |  |  |  |  |
|  | Total | 1.409 E10 | 1028 |  |  |  |  |  |
| Night sleep duration (NSD) | Regression | 5.779 E13 | 6 | 9.631 E12 | 17.055 | <.001 | 0.092 | 0.086 |
|  | Residual Error | 5.715 E14 | 1012 | 5.647 E11 |  |  |  |  |
|  | Total | 6.293 E14 | 1018 |  |  |  |  |  |
| Night sleep efficiency (NSE) | Regression | 1.827 E16 | 4 | 4.568 E15 | 16.727 | <.001 | 0.063 | 0.059 |
|  | Residual Error | 2.728 E17 | 999 | 2.731 E14 |  |  |  |  |
|  | Total | 2.911 E17 | 1003 |  |  |  |  |  |
| Midsleep time (MST) | Regression | 13.594 | 10 | 1.359 | 7.976 | <.001 | 0.074 | 0.065 |
|  | Residual Error | 170.441 | 1000 | 0.170 |  |  |  |  |
|  | Total | 184.035 | 1010 |  |  |  |  |  |
| Wake after sleep onset (WASO) | Regression | 40.181 | 7 | 5.740 | 7.281 | <.001 | 0.047 | 0.040 |
|  | Residual Error | 816.767 | 1036 | 0.788 |  |  |  |  |
|  | Total | 856.949 | 1043 |  |  |  |  |  |
| Daytime Sleepiness (ESS-Score) | Regression | 690.882 | 4 | 172.720 | 15.730 | <.001 | 0.059 | 0.055 |
|  | Residual Error | 11035.088 | 1005 | 10.980 |  |  |  |  |
|  | Total | 11725.969 | 1009 |  |  |  |  |  |
| Subjective Sleep Quality (PSQI Score) | Regression | 4.342 | 6 | 0.724 | 17.224 | <.001 | 0.096 | 0.090 |
|  | Residual Error | 41.088 | 978 | 0.042 |  |  |  |  |
|  | Total | 45.429 | 984 |  |  |  |  |  |

Annotation: SS = sum-of-squares; df = degrees of freedom; MS = Mean square; F = F-ratio; R² = Proportion of explained variance; R²_adj_ = adjusted proportion of explained variance
