# Supplementary figures and images for: Association of serum 25-hydroxyvitamin D concentrations with sleep phenotypes in a German community sample
Source: PLoS One. 2019 Jul 5;14(7):e0219318. doi: 10.1371/journal.pone.0219318 (PMC6611612; doi:10.1371/journal.pone.0219318)

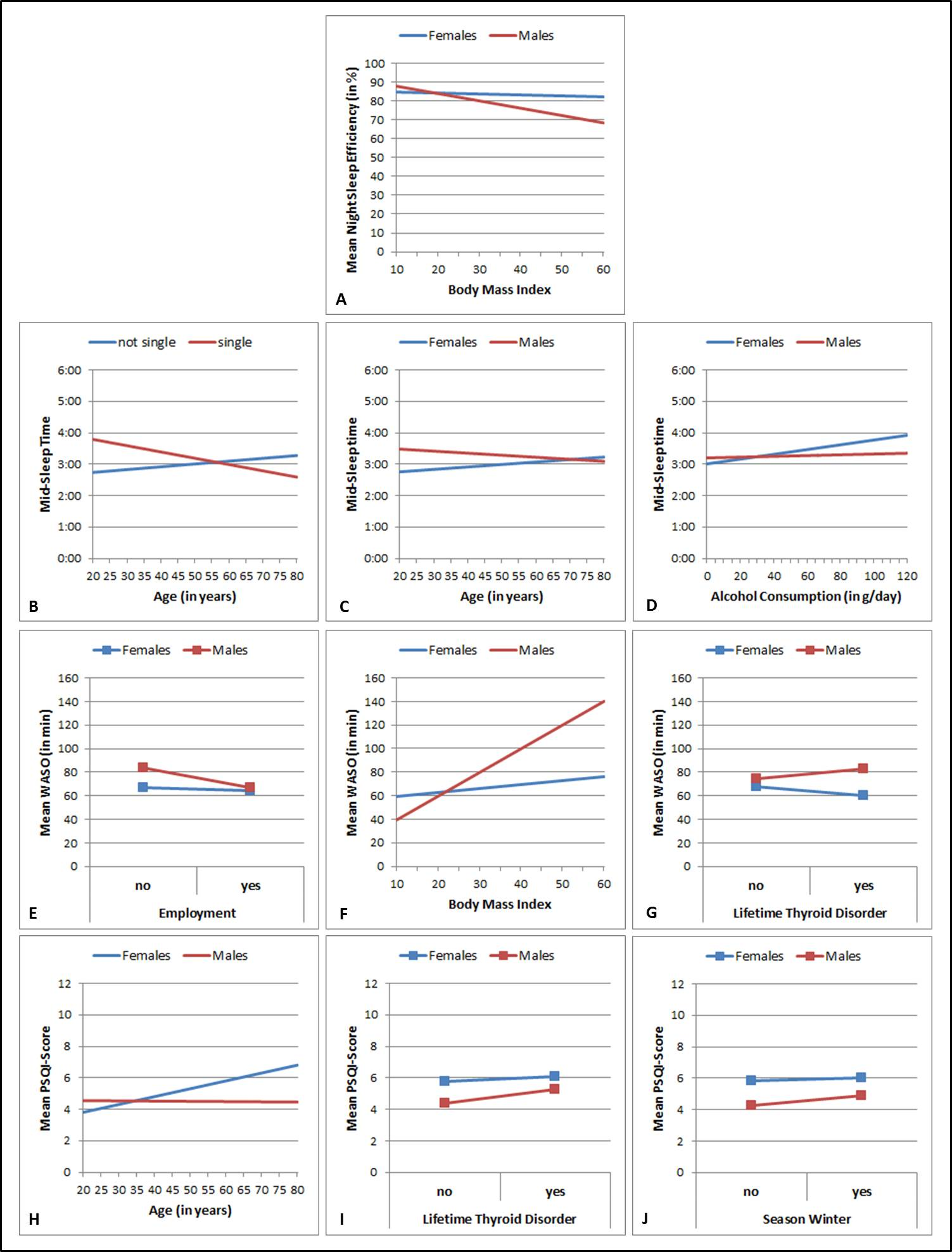

Supplement: S1 Fig — (TIF) [file pone.0219318.s003.tif]
